# Deep Learning Model With Convolutional Neural Network for Detecting and Segmenting Hepatocellular Carcinoma in CT: A Preliminary Study

**DOI:** 10.7759/cureus.21347

**Published:** 2022-01-17

**Authors:** Vo Tan Duc, Phan Cong Chien, Le Duy Mai Huyen, Tran Le Minh Chau, Nguyen Do Trung Chanh, Duong Thi Minh Soan, Hoang Cao Huyen, Huynh Minh Thanh, Le Nguyen Gia Hy, Nguyen Hoang Nam, Mai Thi Tu Uyen, Le Huu Hanh Nhi, Le Huu Nhat Minh

**Affiliations:** 1 Department of Diagnostic Imaging, University Medical Center, Ho Chi Minh City, VNM; 2 Department of Artificial Intelligence - Computer Vision, Vinbrain Company, Hanoi, VNM; 3 Department of Model Development, Vinbrain Company, Hanoi, VNM; 4 Department of of Model Development, Vinbrain Company, Hanoi, VNM; 5 Department of Medical Imaging, University of Medicine and Pharmacy at Ho Chi Minh City, Ho Chi Minh City, VNM; 6 Department of Diagnostic Imaging, Tu Du Hospital, Ho Chi Minh City, VNM; 7 Department of Radiology, Vinmec Healthcare System, Ho Chi Minh City, VNM; 8 Faculty of Medicine, University of Medicine and Pharmacy at Ho Chi Minh City, Ho Chi Minh City, VNM

**Keywords:** computed tomography, dice score, hepatocellular carcinoma, convolutional neural network, deep learning

## Abstract

Introduction

Hepatocellular carcinoma (HCC) is one of the most common malignancies in the world. Early detection and accurate diagnosis of HCC play an important role in patient management. This study aimed to develop a convolutional neural network-based model to identify and segment HCC lesions utilizing dynamic contrast agent-enhanced computed tomography (CT).

Methods

This retrospective study used CT image sets of histopathology-confirmed hepatocellular carcinoma over three phases (arterial, venous, and delayed). The proposed convolutional neural network (CNN) segmentation method was based on the U-Net architecture and trained using the domain adaptation technique. The proposed method was evaluated using 115 liver masses of 110 patients (87 men and 23 women; mean age, 56.9 years ± 11.9 (SD); mean mass size, 6.0 cm ± 3.6). The sensitivity for identifying HCC of the model and Dice score for segmentation of liver masses between radiologists and the CNN model were calculated for the test set.

Results

The sensitivity for HCC identification of the model was 100%. The median Dice score for HCC segmenting between radiologists and the CNN model was 0.81 for the test set.

Conclusion

Deep learning with CNN had high performance in the identification and segmentation of HCC on dynamic CT.

## Introduction

Hepatocellular carcinoma (HCC) is one of the most common primary liver malignancies, more prevalent in regions where chronic viral Hepatitis B and C are prevalent [1,2]. The use of imaging in the diagnosis and treatment of HCC is crucial [3]. This necessitates radiologists to improve their accuracy in both detecting lesions and interpreting images.

The experience of radiologists plays a pivotal role in evaluating images. Furthermore, because HCC is a common development in the context of cirrhosis, doctors are frequently required to interpret many nodules at different stages of HCC development at the time of examination, which may lead to detection flaws [4]. As a result, creating and developing an artificial intelligence model to aid radiologists in recognizing and defining localized liver lesions in patients with HCC risk factors would have numerous medical treatment benefits [5-7].

Artificial intelligence has advanced at a breakneck pace in recent years and is now being extensively used in medicine, particularly in the imaging field. There are a variety of strategies for segmenting the liver and liver tumors; the convolutional neural network (CNN) approach has progressively proven itself in offering more benefits as it can utilize spatial information, providing more accurate segmentation results [7-11]. This research aims to develop a three-dimension (3D) CNN model to identify the location and shape of the primary HCC using computed tomography (CT) images.

## Materials and methods

Study design and data collection

This is a cross-sectional retrospective study involving 110 patients with 115 HCC masses.

Subjects

The medical records and CT imaging studies of 110 patients with 115 HCC lesions were included in this study (107 patients had one, two patients had two tumors, and one patient had four tumors each) and were retrospectively reviewed. The patients were diagnosed with HCC and had operations at the University Medical Center, Ho Chi Minh City, between January 1, 2015, and January 31, 2020. Data regarding demographics and pathologic findings were collected and analyzed.

Inclusion and Exclusion Criteria

At this early stage, we are focusing on developing a CNN model for the detection of HCC. Therefore to ensure the highest accuracy, we only included lesions that underwent resections with clear histopathology results of HCC. We excluded patients that had coexisting non-HCC lesions. In the future, we would expand our research to include late-stage HCCs and widen our selection criteria when our model has shown initial success.

CT Protocol

No patient preparation was required. CT examinations were performed on either 64-slice or 128-slice (SOMATOM® Definition AS+, Siemens Healthineers AG, Erlangen, Germany) scanners with patients in the supine position. The scanning parameters that were used to train the model: CARE Dose4D and CARE kV ON, pitch 1.0, matrix: 512 × 512, recon slice thickness 1 mm, increment 0.8, window level 45 HU, window width 315 HU.

Liver CT examinations were performed with multiphase images (arterial, venous, and delayed phase) after the administration of intravenous contrast material (Xenetix® 300 mgI/ml, Guerbet SA, Villepinte, France; Ultravist® 300 mgI/ml, Bayer AG, Leverkusen, Germany; Omnipaque™ 300 mgI/ml, GE Healthcare Inc., Chicago, United States). The arterial phase was 30-35 seconds after the IV contrast injection and the venous phase was 60-70 seconds; the delayed phase was 180 seconds after the IV contrast injection.

Image Analysis

CT images were interpreted and annotated manually by radiologists who had more than five years of experience in analyzing CT images of liver pathologies on 3D-slicer software. We used the venous phase to measure the tumor diameter. Tumor diameter was recorded as the largest diameter measured on any reconstructed planes. The following major parameters were analyzed: largest diameter of tumor, presence of HCC, the Dice score (demonstrating how well the model performs with the reference of manual tumor segmenting performed by experienced radiologists).

The dataset was randomly divided into a training set (92 patients), and a test set (18 patients). The training set was used to learn the CNN-based model and the test set was used to evaluate the performance of the trained model. 

Proposed CNN-based segmentation method

**Figure 1:**
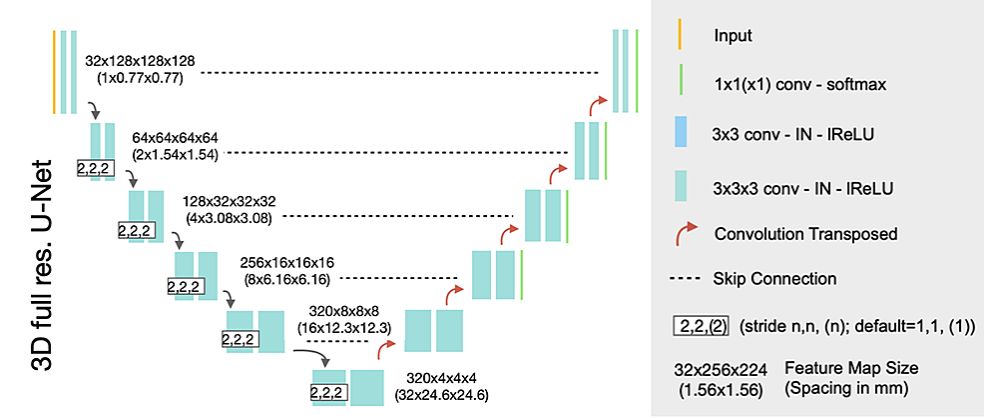
The proposed network for HCC tumor segmentation

The proposed segmentation network is based on U-Net 3D architecture, consisting of two parts: encoder and decoder (Figure 1). The proposed network accepts a single 3D volume and outputs a corresponding 3D HCC segmentation mask. Note that the volume size of the CT is relatively large, e.g. 512×512×551. It is hard to fit into the computational resource, which is limited. Therefore, the CT volume is subdivided into regions of 128×128×128 voxels to feed the proposed model.

The encoder is used to extract the hierarchical characteristic features of the input volume. It contains six blocks, and each block consists of two convolutional layers, each followed by activation layers. The spatial size of each feature map in the next block is reduced by half by setting the stride of two in the first convolutional layer in the block.

The decoder is used to construct the corresponding tumor segmentation mask of the input volume. The decoder includes five blocks, and each block consists of a transposed convolutional layer to double the size of its feature maps, and a convolutional layer. Note that the transposed convolutional or convolutional layer is always followed by an activation layer. The blocks in the decryption part will be symmetric to the blocks in the encryption part. In addition, the network also uses Skip Connections to connect the information of the feature maps of the corresponding blocks between the coder and decoder, retaining more local information when upsampling.

Statistical analysis

Data are shown as mean values ± SDs for normal distribution or median and interquartile range (IQR) if data are not normally distributed. The Dice score measures the overlap between two binary masks. It is the size of the overlap of the two segmentations divided by the total size of the two objects, ranging from 0 (no overlap) to 1 (perfect overlap). Therefore, it indicates the overall segmenting performance of the model. The Dice score was calculated based on the following equation:

$DSC = \frac{2|X\cap Y|}{|X| + |Y|}$

[DSC: Dice similarity coefficient; X: ground truth based on the border of radiologists; Y: the prediction based on the border of the model.]

Spearman's rank-order correlation coefficient was used for Dice score and the tumor diameter. All analyses were performed using Stata Statistical Software: Release 14 (2015, StataCorp LP, College Station, Texas, USA). A p-value < 0.05 was considered significant in all analyses.

Ethical considerations

This study was conducted in the University Medical Center, Ho Chi Minh City, in accordance with the Declaration of Helsinki. The protocol was approved by the Human Research Ethics Committee of the University Medical Center of Ho Chi Minh City. The written informed consent was waived by the Human Research Ethics Committee of the University Medical Center of Ho Chi Minh City. The approval number is 93/GCN-HDDD dated September 17, 2021.

## Results

Participant characteristics

This study includes 110 patients (92 for the training data set and 18 for the testing set), male/female ratio: 87/23 (3.8/1), males 79.1% and females 20.9%. The mean age of this study population is 56.9 ± 11.9 years (26 - 87 years) (Table 1). All patients underwent tumor resection, and all tumors were pathologically proven HCCs.

**Table 1:** Characteristics of participants

Characteristics	Total (n=110)	Training set (n=92)	Test set (n=18)
Age (years), mean ± SD (range)	56.9 ± 11.9 (26 – 87)	57.3 ± 12.4 (26 – 87)	55.1 ± 9.1 (43 – 74)
Gender (Male/Female)	87/23	74/18	13/5

Imaging features of HCC

The mean tumor diameter of both the training set and the test set is 6.0 ± 3.6 cm. Most of the tumors had a diameter of 2 cm or above, accounting for 95.7% (Table 2). Approximately half of the resected tumors (56/115, 48.70%) were recorded as 5 cm or larger based on manual measurement.

**Table 2:** Characteristics of HCC on training and test sets

Characteristics of tumors	Total (n=115)	Training set (n=97)	Test set (n=18)
Size, cm, mean ± SD	6.0 ± 3.6	6.3 ± 3.7	4.5 ± 2.8
≥1 cm and < 2 cm, number (%)	5 (4.3%)	2 (1.7%)	3 (2.6%)
≥2 cm and < 5 cm, number (%)	54 (47.0%)	47 (40.9%)	7 (6.1%)
≥5 cm, number (%)	56 (48.7%)	48 (41.7%)	8 (7.0%)

Dice score

**Figure 2:**
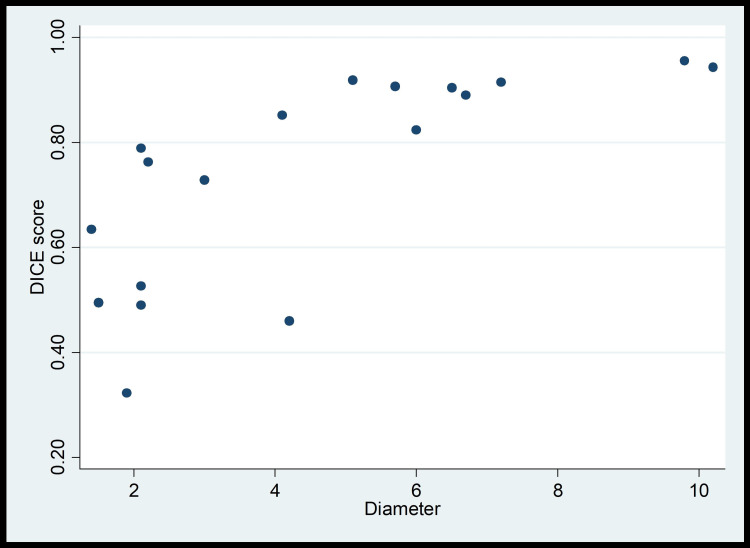
Correlation between tumor size and Dice score

In the test set, the deep-learning model identified HCC in 18/18 cases (100%) with the median Dice score of 0.81 (Interquartile range, 0.53-0.91) (Figure 2).

**Figure 3:**
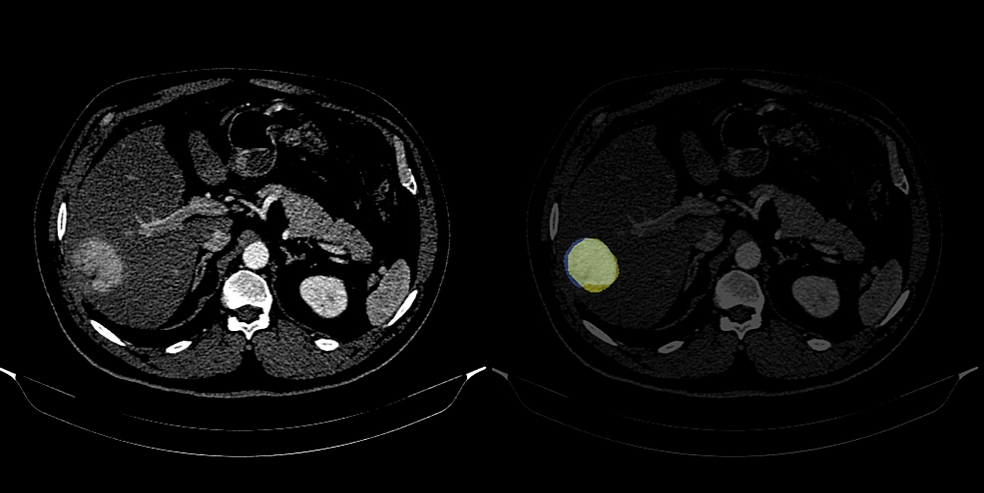
Dice score between radiologists and model Axial CT with contrast injection on arterial phase illustrates a well-differentiated HCC in a 54-year-old male patient; tumor size is 6 cm in diameter. The radiologist border is yellow, the model predict is blue, and the overlap between the doctor and the model is white; the Dice score is 0.9 in this example.

 With well-defined and mass-forming tumors, the Dice score and the diameter were strongly correlated with the Spearman coefficient of 0.82 (p < 0.001) (Figure 3).

## Discussion

In this research, the number of male patients was considerably higher than that of females with an M/F ratio of 3.8/1. This ratio is consistent with the literature and some studies, in which HCC is more common in men than in women, ranging from 2/1 to 4/1 [12-14].

HCC is the most common primary liver malignancy, and one of the most important factors in the treatment strategy and prognosis of patients is the detection of the tumor at an early stage. CT and magnetic resonance imaging (MRI) are the two most used and valuable tools in the diagnosis of HCC, accepted by many guidelines [15-17]. The role of artificial intelligence in diagnostic imaging has been increasingly confirmed through many studies, assisting doctors in detecting, guiding the diagnosis, as well as reducing omission errors [18,19]. For liver lesions, several studies have used CT and MRI images to develop CNN models, and their positive results have shown that artificial intelligence, particularly those based on CNN methods, could help physicians to limit errors and orient the diagnosis [7,20].

This study reported the evaluation of a deep learning-based model to detect HCC based on automatic segmentation of the liver using CNN. The model exhibited a sensitivity of 100% in detecting the tumors on the test set. This remarkable sensitivity can be explained by the fact that most of the patients in our study (83.3%) had tumors larger than 2 cm and were all operated meeting the pathological criteria as a reference standard. Tumors that are less than 1 cm in diameter are notoriously difficult to detect and diagnose based on imaging, ranging from 20-46% with CT and 38-60% with MR imaging [1,21-23]. Kim et al. found that their CNN model had a sensitivity of 84.8% for detecting malignant liver lesions on the test set and that the sensitivity is dependent on the extent of the lesion, with 4.80 false positives per CT scan on the test set [7]. Unlike some other authors, who employed single characteristics in single-phase images, our study and that of Kim et al. provided novel models extracting hemodynamic information from tumors on three phases [24,25]. However, one of the challenges is developing a suitable registration algorithm, which is an area of future research. The research of Kim et al. demonstrated that registration algorithm errors were the source of false positives and negatives, particularly for tiny lesions [7].

The tumor segmentation showed good performance relative to the works of other researchers with a median Dice score of 0.81. Our findings are also very comparable to those of other researchers. For liver tumor segmentation employing a hybrid feature layer, the H-Dense U-Net shows a global Dice value of 0.824 [26]. Using a U-Net variation, attention mechanism, and Skip Connections, AHCNet demonstrated global Dice values of 0.734 for tumor segmentation [27]. Un-Net, which uses an n-fold architecture, has a Dice score of 0.7369 for tumor segmentation [28]. In a study of Ayalew, the Dice score was 0.63 ± 0.02 if the tumors were directly segmented from abdominal CT images, and 0.74 ± 0.02 if tumor segmentation was executed after liver segmentation [5]. In our study, a strong correlation between Dice score and tumor size was observed with the correlation coefficient of 0.82 (p < 0.001). This is comparable to the results of the work of Chlebus et al., in which tumors having the longest diameter of 1 cm or above were detected more reliably than the smaller ones [6]. There were four examples in our analysis where the Dice index was less than 0.5 with the lowest being 0.32. These tumors were smaller than 2 cm in diameter, had abnormal enhancing patterns, and the tumor-liver parenchyma boundaries were not evident. Kim et al. also discovered that lesion size had a substantial impact on sensitivity, with algorithms and physicians alike finding it more difficult to detect smaller lesions [7]. The most common causes of false negatives in the investigation of Kim et al. were atypical enhancement patterns, such as a lack of arterial enhancement and washout in the portal venous and/or delayed phases.

Limitations

There are some drawbacks to this study. First, we only used a dataset from one institute to validate the model. Second, because we only employed CT scanners from one vendor, a decrease in performance may occur when applying the algorithm on different CT systems. Future research will require external validation utilizing a dataset from many institutions with various CT settings. Third, the model was trained to detect only HCC, while liver lesions are highly variable pathology-wise. Finally, in our study, most of the patients had solitary HCC, as we selected resected and pathologically proven HCC for model building and testing at this stage. The tumor group and reference criteria will be broadened in future studies so that the patient group included can be more diverse.

## Conclusions

In conclusion, our preliminary findings indicate that by integrating deep learning and CNN on dynamic contrast-enhanced CT images, detection of HCC can be accomplished with a high degree of sensitivity and a significant Dice score. With the assistance of artificial intelligence, the workloads of radiologists can be significantly reduced, and errors can be substantially limited. Additionally, patients and other physicians can receive imaging reports more rapidly. Patients who are at risk of HCC will be able to benefit from this model if it is widely effectively implemented in multiple centers. However, although the proposed algorithm may be effective in detecting and segmenting HCC, future research is required to achieve satisfactory performance. Specified aspects can be improved and investigated further, such as the CNN model's adaptability to other liver lesions or CT images from different vendors or utilizing artificial intelligence to interpret liver MR images.
